# Demographic transition and the dynamics of measles in six provinces in China: A modeling study

**DOI:** 10.1371/journal.pmed.1002255

**Published:** 2017-04-04

**Authors:** Sheng Li, Chao Ma, Lixin Hao, Qiru Su, Zhijie An, Fubao Ma, Shuyun Xie, Aiqiang Xu, Yanyang Zhang, Zhengrong Ding, Hui Li, Lisa Cairns, Huaqing Wang, Huiming Luo, Ning Wang, Li Li, Matthew J. Ferrari

**Affiliations:** 1 School of Public Health, City University of New York, New York, New York, United States of America; 2 National Immunization Program, China Centers for Disease Control and Prevention, Beijing, China; 3 Jiangsu Provincial Center for Disease Control and Prevention, Nanjing, China; 4 Zhejiang Provincial Center for Disease Control and Prevention, Hangzhou, China; 5 Shandong Provincial Center for Disease Control and Prevention, Jinan, China; 6 Henan Provincial Center for Disease Control and Prevention, Zhengzhou, China; 7 Yunnan Provincial Center for Disease Control and Prevention, Kunming, China; 8 Gansu Provincial Center for Disease Control and Prevention, Lanzhou, China; 9 Global Immunization Division, Centers for Disease Control and Prevention, Atlanta, Georgia, United States of America; 10 Nation Center for AIDS, China Centers for Disease Control and Prevention, Beijing, China; 11 Center for Infectious Disease Dynamics, Pennsylvania State University, University Park, Pennsylvania, United States of America; Imperial College London, UNITED KINGDOM

## Abstract

**Background:**

Industrialization and demographic transition generate nonstationary dynamics in human populations that can affect the transmission and persistence of infectious diseases. Decades of increasing vaccination and development have led to dramatic declines in the global burden of measles, but the virus remains persistent in much of the world. Here we show that a combination of demographic transition, as a result of declining birth rates, and reduced measles prevalence, due to improved vaccination, has shifted the age distribution of susceptibility to measles throughout China.

**Methods and findings:**

We fit a novel time-varying catalytic model to three decades of age-specific measles case reporting in six provinces in China to quantify the change in the age-specific force of infection for measles virus over time. We further quantified the impact of supplemental vaccination campaigns on the reduction of susceptible individuals. The force of infection of measles has declined dramatically (90%–97% reduction in transmission rate) in three industrialized eastern provinces during the last decade, driving a concomitant increase in both the relative proportion and absolute number of adult cases, while three central and western provinces exhibited dynamics consistent with endemic persistence (24%–73% reduction in transmission rate). The reduction in susceptible individuals due to supplemental vaccination campaigns is frequently below the nominal campaign coverage, likely because campaigns necessarily vaccinate those who may already be immune. The impact of these campaigns has significantly improved over time: campaigns prior to 2005 were estimated to have achieved less than 50% reductions in the proportion susceptible in the target age classes, but campaigns from 2005 onwards reduced the susceptible proportion by 32%–87%. A limitation of this study is that it relies on case surveillance, and thus inference may be biased by age-specific variation in measles reporting.

**Conclusions:**

The age distribution of measles cases changes in response to both demographic and vaccination processes. Combining both processes in a novel catalytic model, we illustrate that age-specific incidence patterns reveal regional differences in the progress to measles elimination and the impact of vaccination controls in China. The shift in the age distribution of measles susceptibility in response to demographic and vaccination processes emphasizes the importance of progressive control strategies and measures to evaluate program success that anticipate and react to this transition in observed incidence.

## Abstract 2

Matthew Ferrari and colleagues use a mathematical modelling approach to explore the contribution of both demographic forces and immunization programs to the changing age-distribution of measles cases in China.

## Author summary

**Why was this study done?:**

- Though the incidence of measles has been greatly reduced, achieving elimination remains a significant public health challenge.
- The epidemiology of measles, in particular the age-specific patterns of incidence, is strongly influenced by both demographic rates and vaccination and control efforts.
- China has observed an increase in the number and proportion of measles cases in adults in recent years, a pattern that has been seen in many other countries with successful vaccination programs.
- Our study was designed to quantify the contribution of both demographic forces and immunization programs to the changing age distribution of measles cases in China.

**What did the researchers do and find?:**

- We analyzed 30 years of age-specific measles case reports from six provinces in China using a novel model that accounts for the impact of demographic change and age-targeted vaccination campaigns.
- We found that the increase in adult cases in China is more pronounced in eastern provinces and is consistent with indirect protection from exposure to measles infection in childhood due to herd immunity, but age incidence patterns in the central and western provinces are consistent with continued endemic measles transmission.
- We estimate that while historical supplemental vaccination campaigns resulted in lower immunity than expected based on coverage, recent campaigns have resulted in substantial improvements in population immunity.

**What do these findings mean?:**

- Progress towards measles elimination at the national level is often heterogeneous in space.
- Demographic and age-specific case surveillance data, linked via epidemiological models, can be integrated to provide evaluation of vaccine program performance.

## Introduction

The combination of widespread vaccination, surveillance, and outbreak response has dramatically reduced measles incidence globally [1–3]. Though local elimination of measles was achieved in the Western hemisphere in 2016, the virus remains a major source of vaccine-preventable mortality in many developing countries. In 2012, approximately 122,000 deaths, primarily among children under the age of 5 y, were caused by measles worldwide [4], and resurgent outbreaks have occurred in Europe [5], Africa [6], China [7], and Brazil [8].

Measles has been an exemplar of the interaction between demographic processes and epidemic population dynamics [9–12]. Birth rates have been shown to drive shifts between multi-annual cycles of measles outbreaks [12,13]. Increased vaccination coverage reduces the rate at which new susceptible individuals are added to the population; this has been described as a reduction in the “effective birth rate” as it has been shown to have equivalent consequences to a reduction in the absolute birth rate [12], which may increase the rate of local stochastic fadeout [14] or shift the dynamics from regular multi-annual cycles to episodic outbreaks [12,13]. Declining prevalence, due to vaccination [14,15] or reduced birth rate [16], decreases the per capita infection rate and raises the mean age of infection. The resulting increase in the mean age of the susceptible population may lead to a buildup of susceptible adults that can affect the dynamics of measles outbreaks [17,18]. The same phenomenon has been recognized as a risk factor for congenital rubella syndrome [19,20] when rubella vaccination rates are low.

**Fig 1:**
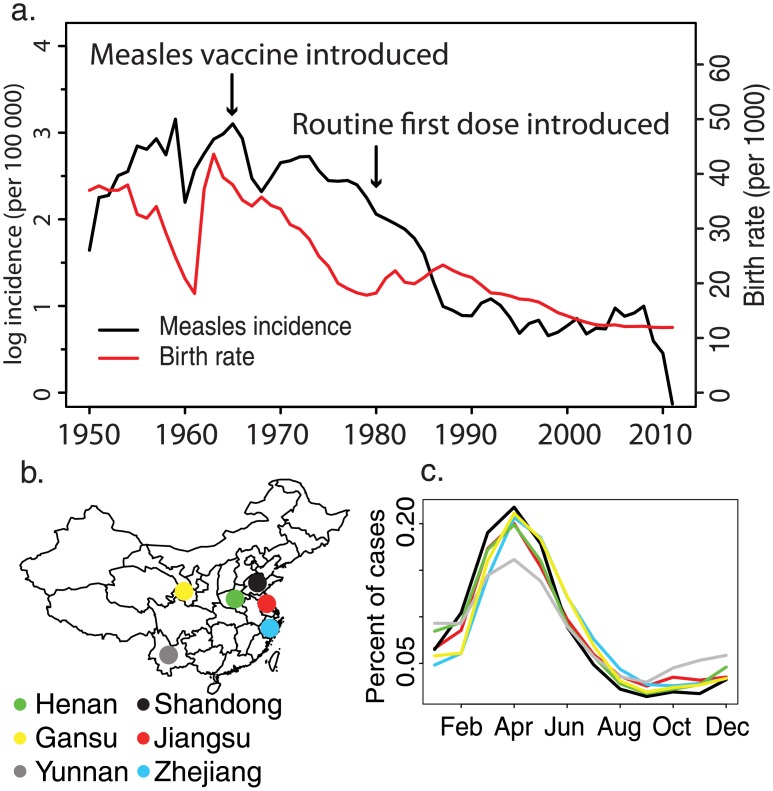
National measles incidence in China. (A) Historical measles incidence (black) and birth rate (red) in China from 1950 to 2011 collected by the China Centers for Disease Control and Prevention. (B) Map of the six focal provinces. (C) Mean percent of measles cases reported in each month for the six focal provinces from 1980 to 2011.

For the past 30 y, China has been experiencing dramatic social and economic development and demographic transition due to the economic reform [21] and the one-child policy [22]. Development and demographic rates vary across China: eastern provinces are more industrialized, urbanized, and densely populated than central and western provinces. Declining birth rates in China (Fig 1A) have changed the age distribution of the population, which may further affect measles transmission [16]; school-aged children, who have been classically associated with measles transmission [23], are underrepresented in China, and the population has been progressively aging since the early 1980s. Measles transmission has been shown to be strongly age specific [24]; however, classic methods for quantifying age-specific transmission require an assumption of stationary dynamics [25]. Neither population dynamics nor epidemic dynamics have been static over time in China. While theory can inform predictions about the response of epidemic dynamics to changes in demography and disease control, evaluating these predictions requires empirical confirmation. Measles persistence in China presents an opportunity to quantify the impact of secular trends in demographics and disease control and to study the dynamics of measles at very low rates of susceptible recruitment [16].

Age-targeted supplementary immunization activities (SIAs) have been a common strategy to improve measles vaccination worldwide [26]. SIAs played an important role in the successful elimination of measles in the Pan American Health Organization region [27]. China has conducted SIAs since 1996; the scale (sub-provincial, provincial, and national) and age targets of SIAs have been highly variable. Quantifying the impact of age-targeted interventions is difficult because the proportion of susceptible persons within the target age classes is rarely known. Using age-specific measles surveillance from China, we quantify both the impact of these interventions on measles immunity at the population scale and the impact on the current age distribution of measles susceptibility.

The goal of this study is to quantify provincial variation in the progress to interruption of measles transmission. For six provinces in China (Fig 1B), we analyze longitudinal, age-specific measles case reports using a novel time-varying catalytic model to estimate change in the age-specific force of infection (FOI) over the last 20 y. In addition, we estimate the impact of age-targeted SIAs on the reduction of population-level measles susceptibility.

## Methods

### Data and study locations

We analyzed measles surveillance data from six provinces in China: three from eastern China (Jiangsu, Shandong, and Zhejiang), two from central China (Henan and Yunnan), and one from western China (Gansu). The eastern provinces are more densely populated (e.g., an average of 1,986 persons/mile^2^ in Jiangsu) and have undergone significant industrial development; the central and western provinces are more agricultural and less densely populated (e.g., an average of 146 persons/mile^2^ in Gansu).

Historical measles and population data were collected retrospectively by China’s national, provincial and prefectural Centers for Disease Control and Prevention (CDC). Prefectural and provincial CDC collected data independently and then submitted them to the national CDC.

The WHO clinical and epidemiological case definitions were applied in all provinces [28]. All recorded cases were either laboratory or epidemiologically confirmed. The total number of cases per year was recorded by age in 1-y intervals for ages 0 to 10 y, and in 5-y intervals for ages 11 to 90 y. Age-specific case reporting began in 1980, 1985, and 1995 for Jiangsu, Shandong, and Henan Provinces, respectively, and in 1990 for Gansu, Yunnan, and Zhejiang Provinces; the last year was 2011 in all provinces. Total non-age-specific cases were collected for each year from 1950 to 1979, and for each month from 1980 to 2011 (S1 Fig), for each province. To correct for potential interannual variation in reporting, a B-spline function was applied to project annual incidence between 1950 and 2011. Prevalence prior to 1950 was assumed to be equal to the level in 1950. The year, target age classes, and target geographic regions for all SIAs were collected from all six provinces (S3 Table).

Population size and age distribution were extracted from China national censuses carried out in 1982, 1990, 2000, and 2010. The population age distribution was reported in the age classes 0–1 y, 2–5 y, and 5-y intervals up to age 90 y. The population size in each age class in noncensus years was projected by linear interpolation.

### The catalytic model

The catalytic model estimates the age-specific FOI by recognizing that the probability of infection at any given age, *a*, can be considered as the probability of remaining susceptible in the interval 0 to *a −* 1 age and the probability of acquiring infection at age *a* [13,23,25]. If the age-specific FOI, θ_*a*_, is constant through time and the prevalence of infection is constant or stationary [29], then the age-specific probability of infection is given by

$$\Gamma_{\left(a\right)}=\left(1-\text{exp}\left(-\theta_{a}\right)\right)*\text{exp}\left(-\overset{a-1}{\underset{y=0}{\sum }}\theta_{y}\right).$$ (1)

The age-specific FOI, θ_*a*_, reflects both the prevalence of infection and the relative age-specific exposure to infection, β_*a*_. Ferrari et al. [13] showed that erratic fluctuations in historical infection prevalence can result in differential historical exposure and thus deviations from the expected proportion susceptible by age under an assumption of constant prevalence (i.e., Eq 1). In China, the prevalence of measles infection has declined dramatically over time due to improved vaccination (Fig 1). Thus, older individuals in the current year will have experienced far greater cumulative FOI by any age *a* than an individual of age *a* in the current year.

The observed vector of age-specific measles cases in year *t*, *I*_*t*_, is of length 25, where each element, *I*_*a*,*t*_, is the number of cases in each age class (ten 1-y age classes for ages 0–10 y and 15 5-y age classes for ages 11–90 y). The vector *I*_*t*_ is modeled as

$$I_{t}\sim \text{multinomial}\left(\sum_{a=1}^{25}I_{a,t},\Omega_{t}\right),$$ (2)

where Ω_*t*_ is a vector of probabilities that sums to 1 and has elements

$$\Omega_{a,t}=\frac{\Lambda_{a,t}}{\sum_{a=0}^{25}\Lambda_{a,t}},$$ (3)

where Λ_*a*,*t*_ is the product of the age-specific probability of infection in year *t*, Γ_*a*,*t*_, and the proportion of the population in age class *a* in year *t*, Φ_*a*,*t*_:

$$\Lambda_{a,t}=\Gamma_{a,t}*\Phi_{a,t}.$$ (4)

The age-specific probability of infection in year *t*, Γ_*a*,*t*_, is modeled as

$$\Gamma_{a,t}=\left(1-\text{exp}\left(-\theta_{a,t}\right)\right)*\left[\text{exp}\left(-\sum_{y=0}^{a-1}\theta_{a-y,t-y}\right)*\prod_{j=1}^{S}\left(1-\psi_{j}A\left(a,j\right)\right)\right],$$ (5)

where θ_*a*,*t*_ is the age-specific FOI, *S* is the number of SIAs, ψ_*j*_ is the unknown effectiveness of the *j*th SIA in each age class (assumed to be the same in all eligible age classes), and A(*a*,*j*) is an indicator function that is 1 if an individual of current age *a* was eligible for the *j*th SIA when it was conducted and 0 otherwise. To account for partial protection of the age class 0–1 y by maternal immunity, we further deprecated by 37.5% [30].

The age-specific FOI θ_*a*,*t*_ is factored as

$$\theta_{a,t}=\beta_{a,t}*P_{t},$$ (6)

where β_*a*,*t*_ is the relative age-specific FOI and *P*_*t*_ is the prevalence of infection, scaled to a maximum of 1. *P*_*t*_ is taken from reported prevalence provided by the China CDC from 1950 to 2011. To correct for potential interannual variation in reporting, a B-spline function was applied to project annual incidence between 1950 and 2011, and we assumed that prevalence prior to 1950 was equal to the 1950 level. We note that while the observed age-specific incidence, *I*_*t*_, in Eq 2 is related to the annual reported prevalence for the years in which the age of cases was reported (post-1980), the likelihood in Eq 2 is based on the relative proportion of cases in each age class in year *t*.

Because we scale the historical prevalence to a maximum of 1, the age-specific relative FOI, β_*a*,*t*_, is indeterminate up to a constant that is related to the reporting rate for measles prevalence. Though the reporting rate is unknown, we assume that reporting is constant over time and the same for all ages; thus, the temporal change in β_*a*,*t*_ reflects decline in FOI over and above that due to the proportional reduction in measles prevalence. See below and S2 and S3 Figs. for analysis of sensitivity to the assumption of constant reporting rate over time.

We allow for the possibility that the relative age-specific FOI is a function of time by fitting a separate β_*a*,*t*_ for each decade (1980–1990, 1991–2000, and 2001–2011) but assuming that β_*a*,*t*_ is constant for all years within each decade; note that *P*_*t*_ changes in each year per the observed prevalence. We model the relative age-specific FOI at time *t*, β_*a*,*t*_, as a basis spline defined by ten unknown parameters: an intercept and nine knots.

The joint likelihood for the time series of annual age-specific case reports within each decade (i.e., β_*a*,*t*_ is constant for all *t*) is the product of the multinomial likelihoods (Eq 1) for each year. We evaluated the joint posterior distribution for the unknown parameters (ten parameters to define the relative age-specific FOI and the impact of each SIA) using a Markov chain Monte Carlo (MCMC) implemented in the statistical software environment R. Markov chains were generated using Gibbs sampling and a Metropolis–Hastings acceptance rule. The MCMC chains were run for 200,000 iterations after a burn-in period of 10,000 iterations. Chains were sampled every 1,000 iterations to avoid autocorrelation; autocorrelation dropped to 0 within 1,000 steps, and effective sample sizes after thinning ranged from 230 to 15,000 for all parameters. We chose noninformative gamma (i.e., strictly positive values) priors for the spline parameters. As reported administrative coverage for SIAs is routinely greater than 90%, we chose informed Beta(9,1) priors for the SIA effectiveness, i.e., the prior assumption is that SIAs reduced susceptible individuals in the target age classes by 90%. Parameter estimates were very robust to the choice of prior (e.g., no difference with a Beta[1,1] prior on the SIA effectiveness); the informative prior was chosen as it resulted in better convergence of the Markov chain. Point estimates are given as the mean of the posterior distribution, and the 95% credible intervals are the 2.5th and 97.5th percentiles of the posterior distribution. Interval estimates of the age-specific FOI function were calculated by taking 2,000 random draws from the posterior distribution of spline parameters, calculating the fitted spline for each, and taking the 5th and 95th quantiles of the resulting splines at each age class.

Inference from long-term trends in case reporting will necessarily be impacted by changes in reporting rates. This has certainly been the case in China, as the health system has developed over the last 30 y, and, recently, between 2000 and 2005, China transitioned from aggregate case reporting to an electronic individual-based reporting system. As a consequence, changes in the absolute numbers of cases are difficult to disentangle from changes in reporting. The model presented here, however, is based on the relative proportion of cases in each age class; the proportion of cases in each age class in the sample of cases reported in each year should be relatively robust to interannual fluctuations or secular trends in reporting, thus allowing us to draw inferences on epidemic dynamics over time without explicitly estimating the reporting rate. To test this assumption, we reconstructed the age-specific case reports for Jiangsu Province under (1) a regime of variable annual reporting with a constant mean rate, with binomial draws from the observed measles cases with an annual probability that is distributed as Beta(9,1), and (2) a linear increase in the annual reporting rate, with binomial draws from the observed measles cases with an annual probability that increases from 0.5 to 1.0. The resulting model fits for the age-specific FOI and the SIA impact are presented in S2 and S3 Figs. The qualitative pattern of the FOI estimates did not differ from the observed data under the two reporting scenarios: the absolute value of FOI declined over time, and the mode shifted to younger ages (S2 Fig). Under linear reporting, the proportional decline in the relative FOI from decade 1 to decade 3 was 10-fold smaller. The estimates of SIA impact were similar for the observed data and the two simulated reporting scenarios: estimated SIA impact increased with time (S3 Fig). The observed data and the variable reporting scenario resulted in indistinguishable estimates of SIA impact. The increasing reporting scenario resulted in elevated estimates of SIA impact, though still well below administrative estimates of vaccination coverage.

We compared our estimate of the susceptible fraction in each age class to independent estimates of age-specific measles seroprevalence from a survey conducted in Jiangsu Province in 2010 [31,32]. The catalytic model fit provides an estimate of the relative FOI, and thus must be scaled to provide an estimate of the absolute seroprevalence. To do so, we scaled the fitted FOI from decade 1 (1980–1990) such that 95% of susceptible individuals would be infected by 15 y of age at the 1980 level of measles prevalence. We then applied this scaling to the estimated FOI from all three decades and estimated the proportion susceptible in each age class in 2010 as the fraction that had not been immunized by routine vaccination (using administrative estimates of first-dose measles vaccine coverage), nor by SIAs (using the estimated vaccine effectiveness from the catalytic model fit), nor by natural infection—i.e., the integral of the scaled, fitted FOI from age 0 until the age in 2010. For infants less than 1 y of age, we assumed that 37.5% of children were protected by maternal immunity [30].

### Fit to Jiangsu data prior to supplementary immunization activities

To rule out the possibility that the temporal trend in FOI is biased by the concentration of SIAs in decade 3 (2001–2011), we fit the time-varying catalytic model to data from Jiangsu Province from the time period 1980–2004, prior to the first provincial SIA in 2005. To illustrate the relative impacts of demographic and prevalence trends on model fit, we fit sub-models that assumed that (1) prevalence and population age distribution held constant, (2) prevalence alone held constant, or (3) population age distribution alone held constant.

## Results

Reported measles incidence in China has declined dramatically over the last 60 y, from 1,000 cases per 100,000 population in the 1950s to 5 cases per 100,000 population in 2010 (Fig 1A). This decline has been accompanied by a similarly large decline in the birth rate and by the introduction and broad-scale distribution of routine measles vaccination (Fig 1A). Despite this large decline in incidence, measles cycles remain strongly annual at the provincial level (Figs 1C and S1), with an annual peak between the 13th and 18th week of the year in all six provinces. Though reported incidence often falls to 0 at the county level, incidence at the province level has remained above 0 from 1980 to 2011 (S1 Fig).

**Fig 2:**
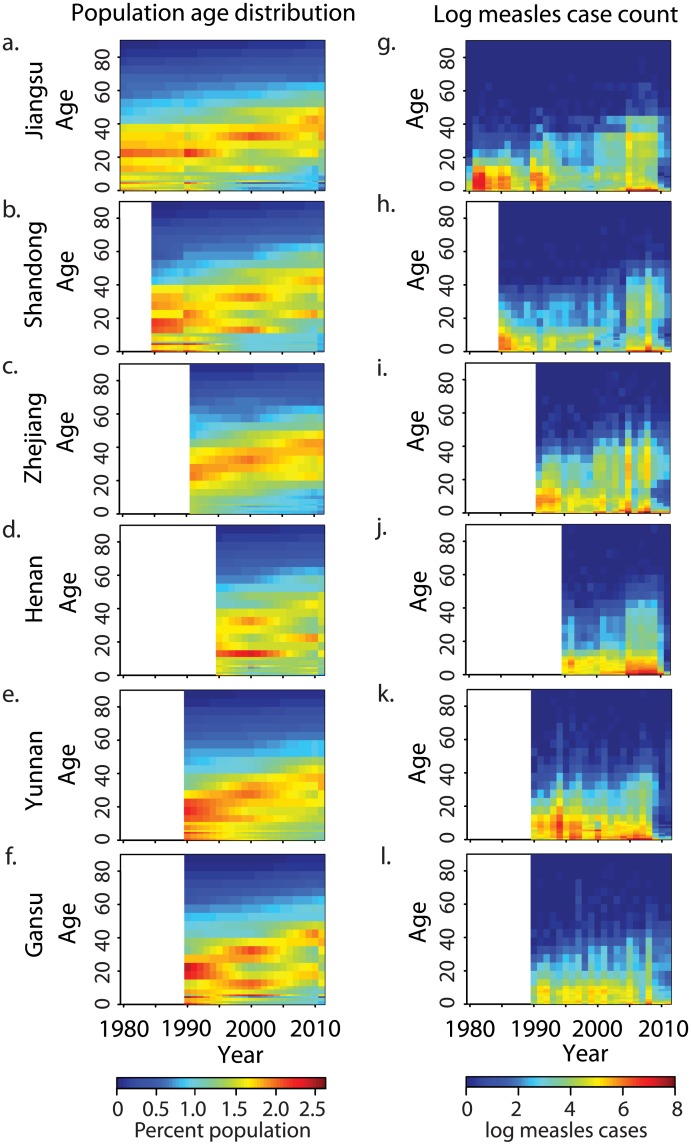
Changing age distribution of population and measles cases in six focal provinces. (A–F) Estimated percent of population in each age class for Jiangsu, Shandong, Zhejiang, Henan, Yunnan, and Gansu Provinces from national census; white indicates years for which no age-specific measles surveillance was available. (G–L) Reported measles cases (log_e_[cases + 1]) per year in each age class for six focal provinces; order and white is as in (A–F).

China has undergone a major demographic transition in the past 30 y due to the one-child policy, instituted in 1979 [22]. The last large cohorts born prior to the one-child policy have not been replaced by new births; this has resulted in an age distribution that is increasingly dominated by adults (Fig 2A–2F). This transition has been less pronounced in Yunnan and Henan Provinces (S1 Table). There has been a concomitant shift in the age distribution of measles cases (Fig 2G–2L; see Table 1 for summary of the data): prior to 1990, the majority of cases were in children younger than 15 y, with a single peak in the 5–15-y age classes; but after 2000, the age distribution of measles cases became bimodal, with peaks in infants (under 2 y) and adults (15–35 y). Although infants under 1 y accounted for the largest number of cases in a single age class from 2000 to 2010, the absolute number of measles cases in individuals greater than 30 y of age has increased over this time.

**Table 1:** Description of age-specific case reporting in six Chinese provinces.

Province	Region	Start year	Range of annual age-specific cases reported		Years of SIAs
			Minimum	Maximum	
Jiangsu	Eastern	1980	354	6,713	2005, 2006, 2007, 2009, 2010, 2011
Zhejiang	Eastern	1991	936	12,782	2005, 2008, 2009, 2010, 2011
Shandong	Eastern	1985	421	9,441	1996, 1999, 2000, 2001, 2004, 2008, 2009, 2010, 2011
Henan	Central	1995	149	9,742	1999, 2000, 2001, 2005, 2011
Yunnan	Central	1990	100	15,793	2000, 2001, 2002, 2003, 2004, 2005, 2006, 2007, 2008, 2010, 2011
Gansu	Western	1990	714	4,217	2002, 2004, 2005, 2008, 2010, 2011

Start year indicates the first year of observed age-specific reporting. All provinces reported age-specific cases through 2011. Years of SIAs indicate years of provincial or sub-provincial supplementary vaccination activities.

### Measles in Jiangsu Province

**Fig 3:**
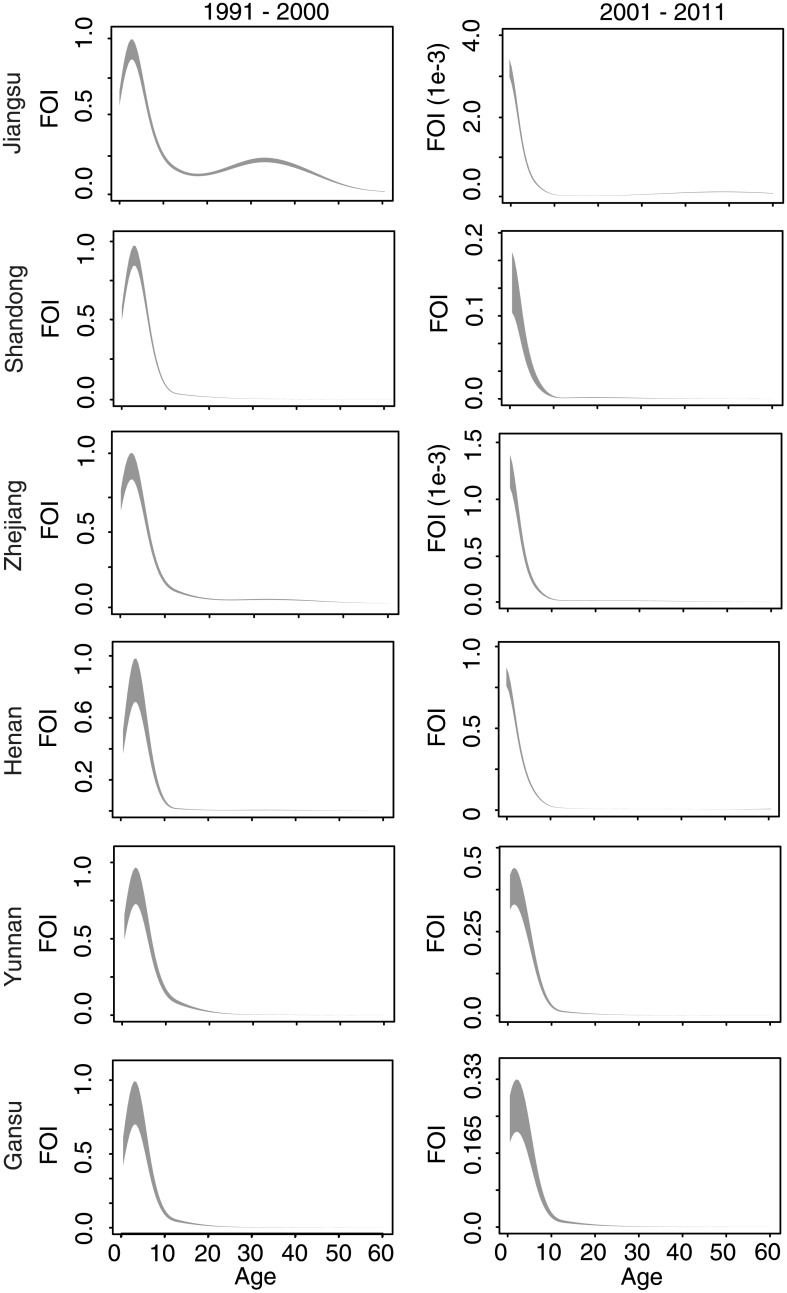
Fitted age-specific force of infection for six focal provinces. Rows indicate Jiangsu, Shandong, Zhejiang, Henan, Yunnan, and Gansu Provinces. Columns indicate model fits for each observed decade. The fitted age-specific force of infection (FOI) is scaled to have a maximum of 1 in the decade 1991–2000 (left column); the FOI in 2001–2011 (right column) in each province is scaled relative to the values in 1991–2000. Grey shading gives the 2.5th and 97.5th percentiles of the posterior distribution of the fitted spline.

We first present an analysis of measles surveillance data from Jiangsu Province, for which the time series is longest and for which we have independent estimates of measles seroprevalence from a field survey in 2010 [31,32]. The fitted catalytic model illustrates two phenomena (Fig 3): a quantitative decline in the absolute value of the FOI, and a shift in the mode of FOI from individuals 5–20 y old (before 2000) to children under 5 y (2001–2011).

When fit to observed age-specific case reporting prior to the first SIA in 2005, the full model, which includes the observed decline in measles prevalence and the shift in population age distribution, produces qualitatively similar results to the model fit to the full time series: a decline in absolute FOI over time and a shift in the mode to younger ages (Figs 3 and S4).

If we assume, in Eq 5, that measles prevalence is constant over historical time, the fitted model for Jiangsu estimates a shift in the mode of the FOI towards adults (S4 Fig). Similarly, if we assume that the age distribution remains flat and constant over time, or that both measles prevalence and population age distribution remain constant over time, the mode of the FOI is estimated to have shifted towards adults in the most recent decade (S4 Fig). Thus, both the observed decline in measles prevalence and the shift in the population age distribution are required to produce the observed FOI pattern over time in the full model.

**Fig 4:**
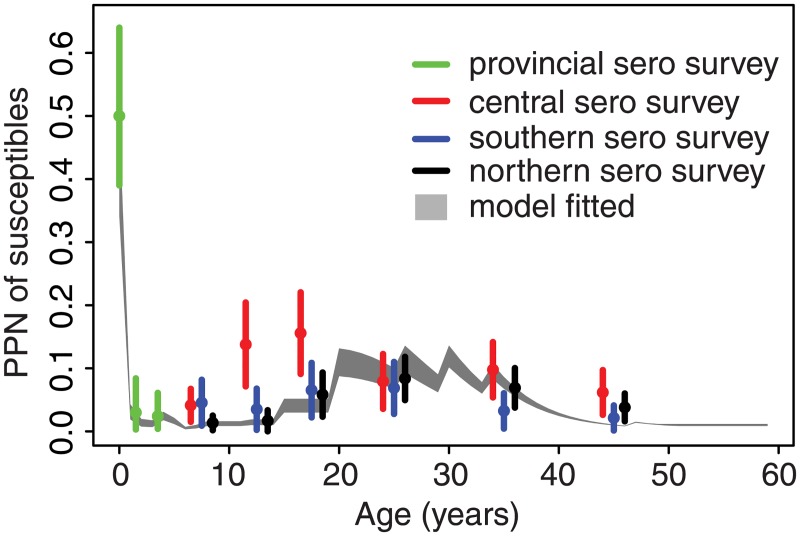
Estimated proportion susceptible in each age class in Jiangsu Province in 2010. Grey shading gives the 95% credible interval for the proportion (PPN) of each age class susceptible to measles as estimated from the catalytic model fit. Circles and vertical bars indicate the point estimates and 95% confidence intervals of the proportion susceptible, by age, for the province and three of its prefectures from a serological (sero) survey conducted in 2010 as reported in Liu et al. [31,32].

The full fitted model broadly predicts the observed seroprevalence from an independent out-of-sample serological survey in Jiangsu [31,32]; estimates across all age classes lie within the confidence bounds from the serological survey from the southern and northern prefectures (Fig 4). The seroprevalence in the central prefecture was higher than the serological testing result within the 15–30-y age classes, which may reflect poor coverage of past SIAs in those age classes in this region.

### Comparative force of infection in six provinces

Comparison of the estimated FOI in all six provinces shows a consistent pattern: FOI declines in overall magnitude with time, and the mode shifts from older to younger individuals (Fig 3). For comparison, we fit a constrained model that assumes that the relative age-specific FOI is constant over time. This constrained model results in a larger sum of squared error (range: 1.1 to 1.2 times greater) between the observed and predicted age distribution of cases than the time-varying model in all six provinces. Further, a model that assumes that relative age-specific FOI is constant over time fails to predict the secondary mode of susceptibility in the 20–40-y age classes seen in the 2010 serological survey in Jiangsu [31,32] (Fig 4). The magnitude of the decline in the FOI was much greater in the three eastern provinces (100- to 1,000-fold decline in Jiangsu, Shandong, and Zhejiang; Fig 3) than in the central and western provinces (0.3- to 0.5-fold decline; Fig 3). This equates to a relative decline in the effective reproductive ratio (*R*_E_) of 97%, 90%, and 95% in Jiangsu, Shandong, and Zhejiang, respectively, and a relative decline in *R*_E_ of 24%, 68%, and 73% in Henan, Yunnan, and Gansu, respectively [33]. This corresponds with a more dramatic increase in the age distribution of cases over time in the eastern provinces (Fig 2).

### Effectiveness of supplementary immunization activities

**Fig 5:**
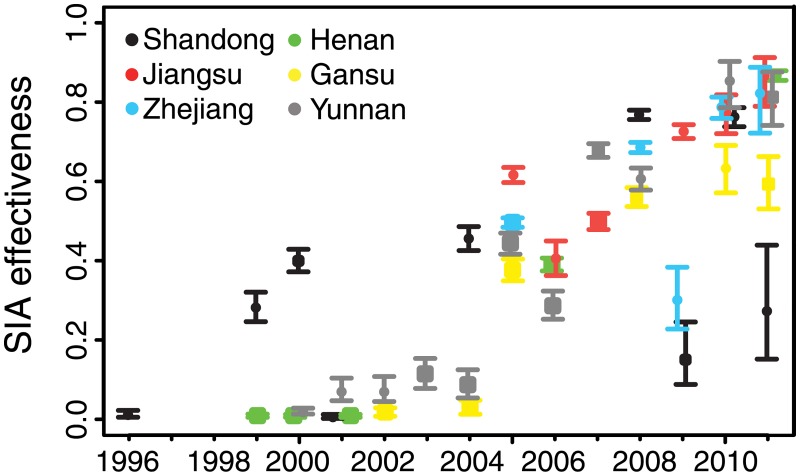
Estimated effectiveness of supplementary immunization activities in six focal provinces. The estimated 95% credible interval for the proportion of susceptible individuals in the targeted age class effectively immunized in each supplementary immunization activity (SIA) is given by the range of the vertical bars. Circles indicate the mean of the posterior distribution for provincial-scale SIAs. Squares indicate the same for sub-provincial SIAs. Overlapping points have been shifted in the *x*-dimension to permit visibility.

For all six provinces, we estimated the effectiveness of SIAs as the proportional reduction in susceptible individuals in the target age classes following the SIA. Since 1996, China has implemented SIAs at the national, provincial, and sub-provincial level. For each SIA, we estimated the proportional reduction in the province-level number of susceptible individuals; thus, sub-provincial SIAs generally are expected to have smaller impact (because they target a subset of the province) than province-wide SIAs. In all provinces, the effectiveness of SIAs tended to increase over time: province-wide SIAs prior to 2005 were estimated to have resulted in 0.5% to 45% reductions in susceptible individuals in the target age classes, while SIAs after 2005 were estimated to have resulted in 32%–87% reductions (Fig 5; S4 Table). Sub-provincial SIAs were generally estimated to have had lower impact than provincial SIAs, relative to the temporal trend (Fig 5; S4 Table). Early SIAs tended to target large age ranges (e.g., up to 14 y), while recent SIAs more frequently targeted children under 6 y; targeting younger ages may account for the increasing trend in effectiveness, as a larger fraction of younger age classes would be expected to be susceptible.

## Discussion

The dependence of epidemic dynamics on demographic rates and disease control activities has been well characterized using theoretical models [10,12,15,34,35]. Here we document the transition in epidemic dynamics in China using highly resolved age-structured measles surveillance data. We have shown that the long-term shift in the age distribution of measles cases in China is consistent with theoretical predictions of the consequences of the decline in overall measles prevalence due to vaccination. Understanding this transition is critical to evaluating the impact of disease control activities, as dynamics (e.g., multi-annual cycles and the age distribution of cases) may change dramatically and could be misinterpreted as a failure of control activities. In particular, the potential role of susceptible adults in measles persistence has been identified as a critical knowledge gap [36]. Understanding the natural demographic processes that contribute to the age distribution of measles cases is critical [14]; spurious attribution of this phenomenon to waning of immunity or vaccine program failure could divert resources (to adult-targeted SIAs or booster doses [36]) from underperforming areas of endemic transition (e.g., the central and western provinces in China). This transition in epidemic dynamics may further lead to unintended consequences of disease control activities, such as the predicted increase in rates of congenital rubella syndrome in populations partially vaccinated with rubella antigen [19], which is consistent with the numerical increase in adult cases of measles in China presented here. Thus, anticipating such age shifts due to vaccination and demographic change is critical to predicting future health outcomes.

Evaluating disease control activities requires methods that predict the expected dynamics in the absence of control [37] and measure impact in terms of a practical outcome. Here we have presented a novel approach for estimating SIA effectiveness that accounts for the temporal shift in the expected age distribution of cases as a function of FOI and demographic change, and quantifies impact in terms of the proportional reduction of susceptible individuals. While vaccination coverage (i.e., the proportion of the population vaccinated) was reported to be high for nearly all of these campaigns, SIAs necessarily vaccinate many individuals who are assumed to be immune because of prior vaccination or natural exposure. Thus, vaccination coverage does not necessarily reflect the reduction in susceptible individuals due to SIAs. Heterogeneity in access to vaccination services may lead to biased vaccination of those who have been previously immunized, leading to a disparity between reported coverage and true impact [38], as seen here. Failure to account for this difference would lead to underestimation of the susceptible population and future outbreak risk.

Our full model incorporated the observed decline in measles prevalence in the model structure. However, we observed that the absolute value of the fitted FOI declined with time, suggesting that the total FOI has declined faster than prevalence. We propose three interpretations to explain this observation. First, an improvement in measles reporting through time would mean that the absolute decline in measles prevalence would be underestimated by the reported data. Thus, the fitted model would be expected to correct for this bias as a further reduction in the absolute magnitude of FOI through time—the sensitivity of these results to variable reporting is presented in S3 Fig. A second explanation is the decline in effective transmission rate as a consequence of declining susceptible recruitment (due to both declining birth rates and increasing vaccination). Earn et al. [12] previously showed the dynamical equivalence of a reduction in birth rate, increase in vaccination rate, and reduction in transmission rate. Since 1980, birth rates in China have declined by half (Fig 1) and vaccination rates have increased 2- to 3-fold [7,39], which reflect a change in FOI consistent with the decline observed in the central and western provinces (Fig 3). Finally, the estimated decline in the absolute value of FOI may be a signature of the nonlinear effect of herd immunity as vaccination increases and prevalence declines. Indeed, the estimated values of FOI in the eastern provinces of Jiangsu, Shandong, and Zhejiang were low enough to indicate that the current levels of infection are insufficient to cause any perceptible depletion of susceptible individuals, i.e., consistent with episodic outbreaks in a regime where the effective reproductive ratio *R*_E_ is below 1. The decline in measles FOI may also be due to improvements in sanitation and health services over time. While we cannot make a definitive estimate of the current value of *R*_E_ without making restrictive assumptions about population mixing, this analysis does indicate that the magnitude of decline in FOI in the three eastern provinces is consistent with a transition from the endemic to episodic regime. The continued observation of cases in these provinces may be due to importation from endemic regions or regional metapopulation dynamics [40], rather than continued chains of local transmission.

The observed shift in the relative FOI to younger age classes was consistent in all six provinces, though more pronounced in the eastern provinces of Jiangsu, Shandong, and Zhejiang. While our analysis did not assume an explicit age-specific mixing contact process (e.g., [41,42]), it is possible that the observed patterns are a result of changes in age-specific mixing over time. However, in the absence of explicit measures of age-specific contact behavior over time, such inference is speculative.

A limitation of this study is its reliance on case reporting. These reports reflect only those cases recorded by the provincial and national surveillance system and may reflect significant underreporting of cases. Age-specific variation in the reporting of cases (e.g., because of less severe symptoms in older individuals) could bias the results. Further, uncertainty in the age-specific population sizes can result in systematic bias in the absolute estimate of FOI [23,25]. In China, internal migration between provinces means that population size and age distribution data from the decadal censuses are only approximate and may introduce bias in the absolute estimates of the FOI [7]. A strength of the approach presented here is that we can project the age distribution of measles immunity using readily available surveillance data; however, we have validated this approach with only a single out-of-sample serological survey, and further validation of these methods with serological surveys is warranted.

Theory has long predicted that epidemic systems may shift dynamical regimes in response to demographic changes and interventions [12]. Rather than take this as a cautionary tale, however, the goal should be to predict these dynamical shifts to evaluate the impact of disease control measures and recommend novel actions. Here we have illustrated the dependence of observable dynamics on secular trends in both demography and disease control and used these trends to develop a novel metric for evaluating vaccination campaign impact. The recent resurgence of measles observed in many countries with long histories of successful control [6,43,44] suggests that prior metrics of program performance may be underestimating susceptible population accumulation and outbreak risk. The methods we present here provide a mechanism to evaluate program performance based on dynamic outcomes, rather than operational measures (i.e., doses delivered).

Changing trends in the age distribution of cases in response to demography and disease control may help to inform improved measles eradication efforts and more powerful measures of progress towards those goals. These generic phenomena are also of relevance for other vaccine-preventable infections for which the risks of adverse health outcomes increase with age, such as varicella and rubella [19,45,46]. Concerns have been raised about the rollout of universal vaccination for both of these viruses as low vaccination coverage may result in a shift of the age distribution towards older individuals, as documented here.

Understanding the transition from the endemic to disease-free equilibrium is critical to planning, evaluating, and monitoring programs to achieve measles elimination. As progress towards this goal is unlikely to be uniform in all areas, identifying local variation in the progression to *R*_E_ < 1 can be useful in prioritizing supplemental control efforts. Here, we have identified an apparent regional disparity in the progression to local elimination. The decline in the magnitude of FOI in the eastern provinces of Jiangsu, Shandong, and Zhejiang is in excess of the decline in prevalence accounted for in the model, and may indicate the nonlinear reduction in FOI through time as a function of herd immunity. The drastic reduction in FOI in these eastern provinces indicates that natural infection is presently removing a trivial proportion of unvaccinated susceptible individuals relative to a regime of endemic transmission, consistent with a transition to subcritical dynamics with *R*_E_ less than 1. The persistence of apparent endemic dynamics in Henan, Gansu, and Yunnan Provinces suggests that national endemicity may be facilitated by local persistence and repeated introductions into regions that are below the endemic threshold [47]. While we found that the large national SIA in 2010 was highly effective at reducing susceptible individuals (Fig 5), subsequent efforts may be more efficient if designed to target endemic regions and limit the migration of infected individuals.

## Supporting information

S1 Fig Measles cases reported each month in each of six focal provinces between 1980 and 2011. (EPS)

S2 Fig Sensitivity of the fitted force of infection in Jiangsu to assumptions about the measles reporting rate. The top row gives the estimated force of infection (FOI) as presented in the main text, the middle row gives estimates assuming that annual measles reporting was variable with constant mean over time, and the bottom row gives estimates assuming that measles reporting increased linearly from 0.5 in 1980 to 1.0 in 2011.
(EPS)

S3 Fig Estimated SIA effectiveness in Jiangsu assuming that reporting rate was constant, randomly varying each year, or increasing linearly from 0.5 in 1980 to 1.0 in 2011. The estimates assuming constant reporting are given in black for comparison.
(EPS)

S4 Fig Fitted age-specific force of infection for Jiangsu Province prior to the first supplementary immunization activity. Rows 1–3 indicate each of the sub-models, and row 4 indicates the fit of the full model. Columns indicate the three decades described in the main text. The last decade includes only the period prior to the first supplementary immunization activity (SIA) in Jiangsu (2001–2004). The sub-models (numbers correspond to rows from top to bottom) are (1) population and measles prevalence assumed to be constant across all time, i.e., all variation in the observed age distribution must be accounted for by the force of infection (FOI) alone; (2) the age-specific population distribution is assumed to change as observed, but measles prevalence is assumed constant; (3) measles prevalence is assumed to decline over time as observed, but the population age distribution is assumed constant; and (4) the full model fit in the main text, but only for the time period 1980–2004.
(EPS)

S1 Table Age class containing the 50th percentile of population in each province for the 1990, 2000, and 2010 censuses. (DOCX)

S2 Table Proportion of population between 6–15 y in each province for the 1990, 2000, and 2010 censuses. (DOCX)

S3 Table The basic characteristics of supplementary immunization activities in six provinces in China. (DOC)

S4 Table The estimated effectiveness, defined as the proportional reduction in susceptible individuals in the target age class, of supplementary immunization activities in six provinces in China. (DOC)

## Abbreviations

CDC: Centers for Disease Control and Prevention
FOI: force of infection
SIA: supplementary immunization activity
