# Supplementary material for: Demographic transition and the dynamics of measles in six provinces in China: A modeling study
Source: PLoS Med. 2017 Apr 4;14(4):e1002255. doi: 10.1371/journal.pmed.1002255 (PMC5380361; doi:10.1371/journal.pmed.1002255)
Supplement: S1 Table — (DOCX) [file pmed.1002255.s005.docx]

**S1 Table.** Age group containing the 50^th^ percentile of population in each province for the 1990, 2000, and 2010 censuses.

| Province | 1990 | 2000 | 2010 |
| --- | --- | --- | --- |
| Jiangsu | 25-30 | 30-35 | 35-40 |
| Zhejiang | 25-30 | 30-35 | 35-40 |
| Shandong | 25-30 | 30-35 | 35-40 |
| Henan | 25-30 | 30-35 | 25-30 |
| Yunnan | 20-25 | 25-30 | 30-35 |
| Gansu | 20-25 | 25-30 | 35-40 |
