# Supplementary material for: Demographic transition and the dynamics of measles in six provinces in China: A modeling study
Source: PLoS Med. 2017 Apr 4;14(4):e1002255. doi: 10.1371/journal.pmed.1002255 (PMC5380361; doi:10.1371/journal.pmed.1002255)
Supplement: S2 Table — (DOCX) [file pmed.1002255.s006.docx]

**S2 Table.**  Proportion of population between 6 -15 years in each province for the 1990, 2000, and 2010 censuses.

| Province | 1990 | 2000 | 2010 |
| --- | --- | --- | --- |
| Jiangsu | 0.14 | 0.15 | 0.08 |
| Zhejiang | 0.15 | 0.13 | 0.09 |
| Shandong | 0.16 | 0.16 | 0.10 |
| Henan | 0.18 | 0.20 | 0.13 |
| Yunnan | 0.21 | 0.18 | 0.14 |
| Gansu | 0.17 | 0.20 | 0.13 |
