# Supplementary material for: Demographic transition and the dynamics of measles in six provinces in China: A modeling study
Source: PLoS Med. 2017 Apr 4;14(4):e1002255. doi: 10.1371/journal.pmed.1002255 (PMC5380361; doi:10.1371/journal.pmed.1002255)
Supplement: S3 Table — (DOC) [file pmed.1002255.s007.doc]

**S3 Table. The basic characteristics of SIAs in 6 provinces in China**

| **Province** | **SIA #** | **Year** | **Target age group** | **Sub- or full-province** |
| --- | --- | --- | --- | --- |
| Jiangsu | 1 | 2005 | < 7 year | Full |
| Jiangsu | 2 | 2006 | 1.5- 4 year | Full |
| Jiangsu | 3 | 2007 | 8 month – 14 year | Sub |
| Jiangsu | 4 | 2009 | 8 month – 14 year | Full |
| Jiangsu | 5 | 2010 | 8 month – 4 year | Full |
| Jiangsu | 6 | 2011 | 8 month – 4 year | Full |
| Zhejiang | 1 | 2005 | 8 month – 7 year | Sub |
| Zhejiang | 2 | 2008 | 8 month – 6 year | Full |
| Zhejiang | 3 | 2009 | 15 – 19 year | Full |
| Zhejiang | 4 | 2010 | 8 month – 4 year | Full |
| Zhejiang | 5 | 2011 | 15 - 16 year | Full |
| Shandong | 1 | 1996 | 1.5 – 4.5 year | Full |
| Shandong | 2 | 1999 | 8 month – 7 year | Full |
| Shandong | 3 | 2000 | 8 month – 4 year | Sub |
| Shandong | 4 | 2001 | 7 – 14 year | Sub |
| Shandong | 5 | 2004 | 8 month – 6 year | Full |
| Shandong | 6 | 2008 | 8 month – 6 year | Full |
| Shandong | 7 | 2009 | 7 – 14 year | Sub |
| Shandong | 8 | 2010 | 8 month – 6 year | Full |
| Shandong | 9 | 2011 | 19 – 23 year | Full |
| Henan | 1 | 1999 | 8 month – 14 year | Sub |
| Henan | 2 | 2000 | 8 month – 14 year | Sub |
| Henan | 3 | 2001 | 8 month – 14 year | Sub |
| Henan | 4 | 2005 | 8 month – 14 year | Sub |
| Henan | 5 | 2010 | 8 month – 14 year | Full |
| Yunnan | 1 | 2000 | 8 month – 14 year | Full |
| Yunnan | 2 | 2001 | 8 month – 14 year | Full |
| Yunnan | 3 | 2002 | 8 month – 14 year | Full |
| Yunnan | 4 | 2003 | 8 month – 14 year | Sub |
| Yunnan | 5 | 2004 | 8 month – 14 year | Sub |
| Yunnan | 6 | 2005 | 8 month – 14 year | Sub |
| Yunnan | 7 | 2006 | 8 month – 14 year | Sub |
| Yunnan | 8 | 2007 | 8 month – 6 year | Sub |
| Yunnan | 9 | 2008 | 8 month – 14 year | Full |
| Yunnan | 10 | 2010 | 8 month – 6 year | Full |
| Yunnan | 11 | 2011 | 8 month – 17 year | Sub |
| Gansu | 1 | 2002 | 8 month – 14 year | Sub |
| Gansu | 2 | 2004 | 8 month – 12 year | Sub |
| Gansu | 3 | 2005 | 8 month – 12 year | Sub |
| Gansu | 4 | 2008 | 8 month – 14 year | Sub |
| Gansu | 5 | 2010 | 8 month – 4 year | Full |
| Gansu | 6 | 2011 | 8 month – 14 year | Sub |
