# Supplementary material for: Demographic transition and the dynamics of measles in six provinces in China: A modeling study
Source: PLoS Med. 2017 Apr 4;14(4):e1002255. doi: 10.1371/journal.pmed.1002255 (PMC5380361; doi:10.1371/journal.pmed.1002255)
Supplement: S4 Table — (DOC) [file pmed.1002255.s008.doc]

**S4 Table. The estimated effectiveness, defined as the proportional reduction of susceptible individuals in the target age group, of SIAs in 6 provinces in China.**

| **Province** | **SIA #** | **Year** | **Mean SIA effectiveness** | **95% CI of SIA effectiveness** | |
| --- | --- | --- | --- | --- | --- |
|  |  |  |  | **2.5%** | **97.5%** |
| Jiangsu | 1 | 2005 | 0.624 | 0.605 | 0.643 |
| Jiangsu | 2 | 2006 | 0.413 | 0.370 | 0.457 |
| Jiangsu | 3 | 2007 | 0.507 | 0.486 | 0.527 |
| Jiangsu | 4 | 2009 | 0.734 | 0.716 | 0.751 |
| Jiangsu | 5 | 2010 | 0.781 | 0.728 | 0.826 |
| Jiangsu | 6 | 2011 | 0.870 | 0.797 | 0.920 |
| Zhejiang | 1 | 2005 | 0.519 | 0.507 | 0.531 |
| Zhejiang | 2 | 2008 | 0.709 | 0.696 | 0.721 |
| Zhejiang | 3 | 2009 | 0.324 | 0.251 | 0.407 |
| Zhejiang | 4 | 2010 | 0.810 | 0.782 | 0.836 |
| Zhejiang | 5 | 2011 | 0.845 | 0.745 | 0.911 |
| Shandong | 1 | 1996 | 0.012 | 0.006 | 0.023 |
| Shandong | 2 | 1999 | 0.283 | 0.247 | 0.321 |
| Shandong | 3 | 2000 | 0.401 | 0.373 | 0.430 |
| Shandong | 4 | 2001 | 0.006 | 0.003 | 0.013 |
| Shandong | 5 | 2004 | 0.456 | 0.426 | 0.487 |
| Shandong | 6 | 2008 | 0.769 | 0.757 | 0.780 |
| Shandong | 7 | 2009 | 0.151 | 0.089 | 0.246 |
| Shandong | 8 | 2010 | 0.764 | 0.739 | 0.787 |
| Shandong | 9 | 2011 | 0.273 | 0.153 | 0.440 |
| Henan | 1 | 1999 | 0.005 | 0.002 | 0.011 |
| Henan | 2 | 2000 | 0.005 | 0.002 | 0.010 |
| Henan | 3 | 2001 | 0.005 | 0.002 | 0.010 |
| Henan | 4 | 2005 | 0.388 | 0.372 | 0.404 |
| Henan | 5 | 2010 | 0.865 | 0.852 | 0.876 |
| Yunnan | 1 | 2000 | 0.010 | 0.005 | 0.020 |
| Yunnan | 2 | 2001 | 0.061 | 0.038 | 0.095 |
| Yunnan | 3 | 2002 | 0.061 | 0.036 | 0.099 |
| Yunnan | 4 | 2003 | 0.101 | 0.069 | 0.145 |
| Yunnan | 5 | 2004 | 0.073 | 0.045 | 0.116 |
| Yunnan | 6 | 2005 | 0.434 | 0.408 | 0.461 |
| Yunnan | 7 | 2006 | 0.278 | 0.244 | 0.315 |
| Yunnan | 8 | 2007 | 0.670 | 0.652 | 0.687 |
| Yunnan | 9 | 2008 | 0.598 | 0.570 | 0.625 |
| Yunnan | 10 | 2010 | 0.844 | 0.777 | 0.894 |
| Yunnan | 11 | 2011 | 0.809 | 0.733 | 0.867 |
| Gansu | 1 | 2002 | 0.014 | 0.007 | 0.029 |
| Gansu | 2 | 2004 | 0.024 | 0.012 | 0.048 |
| Gansu | 3 | 2005 | 0.376 | 0.349 | 0.404 |
| Gansu | 4 | 2008 | 0.560 | 0.536 | 0.584 |
| Gansu | 5 | 2010 | 0.630 | 0.568 | 0.688 |
| Gansu | 6 | 2011 | 0.598 | 0.530 | 0.662 |
